# Retraction Note: An optimal feed-forward artificial neural network model and a new empirical correlation for prediction of the relative viscosity of Al_2_O_3_-engine oil nanofluid

**DOI:** 10.1038/s41598-026-62698-7

**Published:** 2026-07-15

**Authors:** Mohammad Hemmat Esfe, Davood Toghraie

**Affiliations:** 1 Department of Mechanical Engineering, Imam Hossein University, Tehran, Iran; 2 Department of Mechanical Engineering, Khomeinishahr Branch, Islamic Azad University, Khomeinishahr, Iran

Retraction of: *Scientific Reports* 10.1038/s41598-021-96594-z, published online 23 August 2021

The Editors have retracted this Article.

After publication, concerns were raised about the data availability of this Article and the results presented in Fig 10. Although, these concerns were addressed by the raw data and code provided by the Authors, further investigations by the Editors revealed the presence of multiple irrelevant references in this Article (References 41-48). Upon request, the Authors could not provide a satisfactory response to these additional concerns.

The Editors therefore no longer have confidence in the reliability of the content of this Article.

The Authors did not state whether they agree to this retraction.

